# Rotational Triboelectric Nanogenerator with Machine Learning for Monitoring Speed

**DOI:** 10.3390/s25082533

**Published:** 2025-04-17

**Authors:** Chun Zhang, Junjie Liu, Yilin Shao, Xingyi Ni, Jiaheng Xie, Hongchun Luo, Tao Yang

**Affiliations:** 1 School of Science, Xi’an Shiyou University, Xi’an 710065, China; chunz@xsyu.edu.cn (C.Z.);; 2 Lanzhou Center for Theoretical Physics, Key Laboratory of Theoretical Physics of Gansu Province, Lanzhou University, Lanzhou 730000, China; 3 School of Computer Science, Xi’an Shiyou University, Xi’an 710065, China; 4 Faculty of Science, Yibin University, Yibin 644007, China; 5 Department of Engineering Mechanics, Northwestern Polytechnical University, Xi’an 710072, China

**Keywords:** triboelectric nanogenerator, energy harvesting, machine learning, rotational speed

## Abstract

The triboelectric nanogenerator (TENG) is an efficient mechanical energy harvesting device that exhibits excellent performance in the fields of micro-nano energy harvesting and self-powered sensing. In practical application scenarios, it is very important to monitor the speed of rotational machinery in real time. In order to monitor a wider range of rotational speeds, the TENG based on a machine learning algorithm is designed in this paper. The peak power of the TENG reaches a maximum of 6.6 mW and can instantly light up 65 LEDs connected in series. The results show that machine learning can detect speed, greatly improving the speed detection range. The neural network is trained and tested based on the collected electrical signals at different speeds so as to monitor the health of the machine. For the analysis of the collected experimental data, normalization data and a more practical label assignment method of Gaussian soft coding were considered. The study found that after data normalization, the classification prediction accuracy for different speeds is above 0.9, and the prediction results are stable and efficient. Therefore, the machine learning prediction model for speed monitoring proposed by us can be applied to the early warning and monitoring of rotating machinery speed in actual engineering projects.

## 1. Introduction

The monitoring of mechanical equipment speed is of great significance in fields such as industry and transportation [1,2,3]. For example, during the rotation of oil drilling machines, accurate speed monitoring can help prevent the drill bit from overheating and avoid drilling accidents, ensuring the continuity and safety of drilling operations [4,5,6,7]. In addition, in the safe flight of aircraft, engine speed monitoring is also one of the key factors which can help detect whether the engine is working in its best state and prevent potential failures [8,9]. In automobile engines, speed monitoring can optimize fuel efficiency and reduce emissions, as well as improve vehicle performance [10,11]. Therefore, the monitoring of motor rotation is used in industrial automation to ensure the smooth operation of the production line. The TENG is an efficient energy harvesting technology that converts mechanical energy into electrical energy through the triboelectric and electrostatic induction coupling effect. It can not only provide power for small electronic devices but also be used to monitor and sense the operating status of mechanical systems [12,13,14,15]. Among various forms of energy harvesting and sensing applications, the TENG has gradually attracted widespread attention due to its high energy conversion efficiency, low cost, and environmental friendliness [16,17,18,19,20]. In recent years, with the rapid development of the TENG, it has been widely used, especially in the energy field [21,22,23], as well as in important fields such as emerging medical care and artificial intelligence agriculture [24,25,26,27]. Additionally, recent studies have applied the TENG to emerging fields such as high-voltage triboelectric-driven amphibious soft robots [28]. However, the output performance of the TENG in applications of these scenarios is affected by many factors, among which the rotation speed of mechanical equipment is an important parameter.

However, the output characteristics of the TENG are relatively complex, and traditional speed sensors based on the TENG can only monitor the speed within a certain speed range. In addition, traditional methods based on physical models make it difficult to achieve high-precision prediction of its speed. In recent years, with the rapid development of artificial intelligence technology, machine learning algorithms have made significant progress in processing complex systems [29,30,31]. In particular, data-driven prediction methods provide new ideas for solving this problem [32,33,34,35]. Compared with traditional methods, machine learning methods can not only handle complex nonlinear relationships but also have strong adaptability and generalization capabilities [36,37,38].

In particular, machine learning can not only analyze and process the electrical signals collected by the TENG but also establish a complex working mechanism between the independent variables and dependent variables of the nanogenerator. This allows for the prediction and optimization of nano energy, overcoming technical limitations in areas like mechanism analysis, prototype design, fabrication and testing, and performance evaluation of the TENG [39,40,41,42]. Based on the electrical signal of the TENG to monitor the driver’s steering action, Zhang et al. [43] proposed a machine learning algorithm to prevent traffic accidents. Das et al. [44] suggested attaching multiple flexible TENG devices made of nanocomposite materials to the human body, using machine learning to accurately track the nature of body movements. Scholars have conducted extensive research the applications of machine learning combined with TENGs. However, there are few reports on the use of machine learning algorithms to automatically establish a model of the relationship between TENG output signals and machine speed based on experimental data. This gap highlights the need for further research on using the TENG to achieve accurate speed prediction.

In this paper, a machine learning approach was introduced into a rotational TENG, enabling an accurate prediction speed of rotational machinery. In Section 2, the basic working principle of the rotational TENG and its output characteristics are introduced, along with a description of the experimental data collection process. Section 3 discusses the experimental results and the machine learning process based on TENG electrical signal prediction in detail. It also presents a comparative analysis of the application results of these algorithms in speed prediction. In Section 4, the prediction accuracy and applicability of the proposed methods are evaluated through multiple computations, and the potential for a further application of machine learning technology in the TENG field is discussed. Finally, we present some experimental and machine learning details.

## 2. Model and Power Generation Principle

In order to use machine learning based on the electrical signal of the TENG to monitor the speed of rotational machinery, the output working mechanism and application scenarios of TENG electrical signals are discussed in this section.

**Figure 1:**
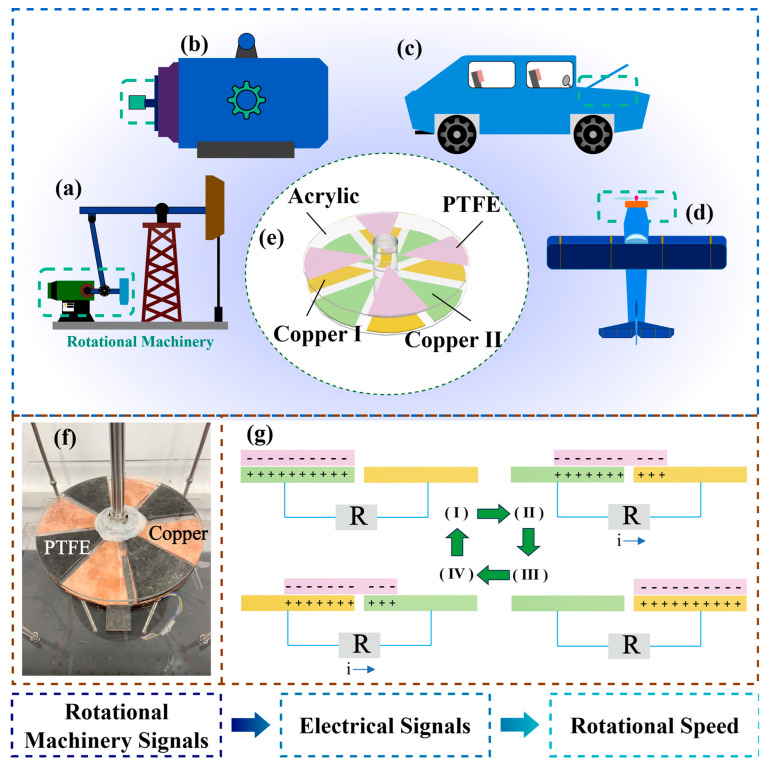
Application, structure diagram, and working principle of TENG. (**a**–**d**) Application of TENG in oil drilling platforms, aerospace vehicles, and rotating machines. (**e**) Structural design diagram of TENG. (**f**) Experimental prototype of TENG. (**g**) Working principle diagram of TENG.

The application scenarios, structural details, and the principle of rotational speed prediction using the TENG are shown in Figure 1. Rotating structures are widely present in machinery such as oil drilling platforms, aircraft, and vehicles (see Figure 1a–d). In these applications, the real-time monitoring of rotational speed is often necessary. Rotational machinery typically generates vibrations during operation, which can reduce the sensitivity and lifespan of traditional speed sensors. Moreover, conventional sensors tend to have complex structures and large sizes, making installation difficult and increasing costs. Therefore, this paper proposes a TENG-based device for speed measurement, shown in Figure 1e, which features high sensitivity, a simple structure, and compact size. The TENG is composed of acrylic plates, polytetrafluoroethylene (PTFE), and copper films, with the adjacent copper films represented as green and yellow for clarity in the explanation of the working principle.

**Figure 2:**
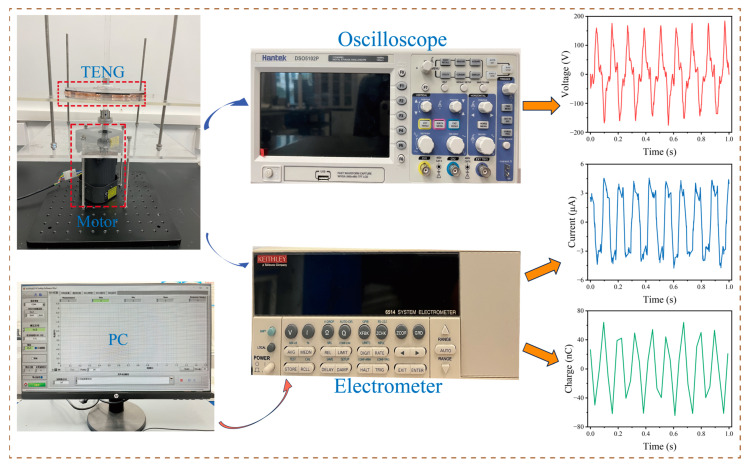
Experimental equipment diagram of TENG.

The experimental prototype is illustrated in Figure 1f. This experimental device consists of a stator, a rotor, a four-segment copper electrode grid, a PTFE friction material, acrylic plates, and supporting bearings. The four-segment copper grid serves as the electrode, attached to the acrylic plate as the stator, while the four-segment PTFE is attached to the acrylic plate as the rotor. The working principle of the device is shown in Figure 1g. When the left copper film (green) contacts the PTFE, the PTFE, being more electronegative, acquires a negative charge, while the left copper film becomes positively charged (corresponding to position I). As the PTFE rotates counterclockwise, the positive charge on the left copper film flows toward the right copper film, generating a rightward current to balance the potential difference caused by the movement of the PTFE (corresponding to position II). When the PTFE reaches the right copper film (yellow), both the PTFE and the right copper film have positive charges, and the system is in electrostatic balance (corresponding to position III). As the PTFE continues to rotate counterclockwise, the positive charge on the right copper film flows to the next copper film (green), generating a rightward current again (corresponding to position IV). When the PTFE completes its movement to the next copper film (corresponding to position I), one rotation cycle is complete. Thus, as the TENG rotates, driven by the motor, it generates alternating current (AC) signals. Figure 2 shows the equipment used in the experiment. The motor generates a fixed rotational speed, which is transmitted to the TENG via the rotating shaft. A static electrometer and an oscilloscope are used to collect the electrical signals from the TENG. When the engine rotates, the vibration of the rotor generates mechanical signals. As these signals are transmitted to the TENG, they are converted into electrical signals. These electrical signals are then sent to a computer, where machine learning analysis is conducted to infer the engine’s rotational speed.

## 3. Results and Discussion

The collected voltage signal contains enough characteristic information reflecting the state of the TENG [45,46,47,48]. Therefore, in order to more comprehensively extract the characteristic information reflecting the state of the TENG, this work adopts the assignment method of Gaussian soft label machine learning to establish a nonlinear relationship by analyzing the collected voltage signals. Then, the corresponding rotational speed is predicted by the voltage. The whole process consists of four parts. First, the TENG platform is built through experiments, then the data signal is collected. Next, the data are input into the neural network we constructed for training. Finally, the prediction result is output.

### 3.1. Electrical Output Performance and Early Warning

**Figure 3:**
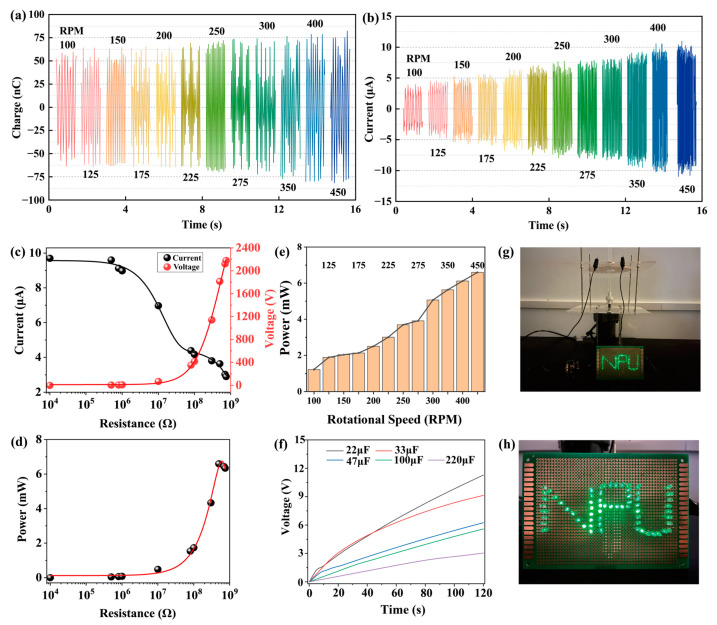
The output and application of TENG. (**a**) Quantity of charge transferred by TENG at various rotational speeds. (**b**) Short-circuit current of TENG at different rotational speeds. (**c**) Short-circuit current and output voltage under different load resistances. (**d**) Power under varying load resistances. (**e**) Peak power at different rotational speeds. (**f**) Voltage curves for TENG charging capacitors of varying sizes. (**g**,**h**) TENG illuminating LED lights.

This study applies machine learning algorithms to predict the rotational speed of a TENG. As an intelligent data-driven approach, good predictive performance relies on relevant data that reflect the operational status of the TENG. The voltage signal, as the output of the TENG, contains sufficient information to indicate its operational state [48,49]. When capturing the output signals from the TENG, the motor is adjusted to generate the corresponding rotational speed for optimal TENG operation. At each designated speed, an electrostatic voltmeter is utilized to collect short-circuit current and transferred charge data, while a digital oscilloscope captures the output voltage, all for a duration of 1 s. The charge transferred by the TENG at varying speeds is illustrated in Figure 3a, with charges at 100, 125, 150, 175, 200, 225, 250, 275, 300, 325, 350, 375, 400, 425, and 450 rpm being 59.6, 64.1, 64.4, 65.3, 65.5, 69.2, 72.2, 73.6, 73.9, 74.5, 76.9, 78.1, 80.0, 81.1, and 82.2 nC, respectively. The corresponding short-circuit currents, depicted in Figure 3b, are recorded as 4.2, 4.8, 5.6, 5.9, 6.8, 7.4, 7.9, 8.1, 8.3, 9.0, 9.5, 10.2, 10.6, 10.7, and 11.0 μA at the same rotational speeds. Figure 3c shows the short-circuit current and output voltage of the TENG under different load resistances, with a corresponding short-circuit current of 9.7 μA and an open-circuit voltage of 2180 V. In Figure 3d, the peak power of the TENG reaches a maximum of 6.6 mW when the load resistance is set to 500 MΩ. In Figure 3e, the peak power of the TENG at a load resistance of 500 MΩ across rotational speeds of 100, 125, 150, 175, 200, 225, 250, 275, 300, 325, 350, 375, 400, 425, and 450 rpm is recorded as 1.2, 1.9, 2.0, 2.1, 2.5, 3.0, 3.7, 3.9, 5.1, 5.5, 5.6, 6.0, 6.1, 6.5, and 6.6 mW, respectively. Figure 3f illustrates the voltage characteristic curves of the TENG charging capacitors of 22, 33, 46, 100, and 220 μF at a rotational speed of 450 rpm. After a charging time of 120 s, the corresponding voltages across the capacitors are 11.3, 9.1, 6.2, 5.6, and 3.0 V. Figure 3g,h shows photos of the TENG illuminating 65 LED lights. When the TENG operates, the NPU light assembly composed of 65 LEDs is instantaneously illuminated in series. On one hand, the TENG converts mechanical energy into electrical energy, providing power for small electronic devices. On the other hand, this work aims to explore the application of TENG-based electrical signals for monitoring rotational mechanical speed.

### 3.2. Framework for Algorithms in Machine Learning

**Figure 4:**
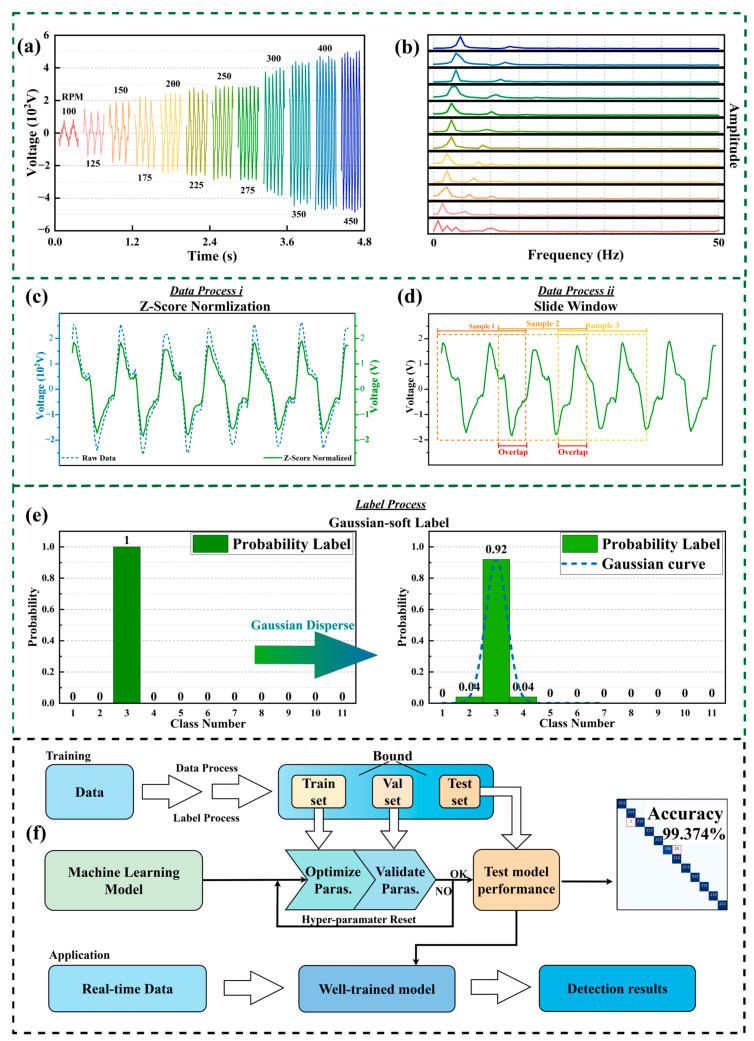
Data processing and machine learning flow chart. (**a**) Raw voltage signals collected by the TENG at different rotational speeds. (**b**) The frequency domain information is presented after performing a Fourier transform on 128-length segments of the original data at each speed. From top to bottom, the rotational speeds are 450 to 100 rpm, corresponding to Figure (**a**). (**c**,**d**) Data processing procedure. First, the experimental raw data are collected, followed by normalization. Finally, the data are segmented into equal lengths using a sliding window. (**e**) Schematic illustration of the operation details of the Gaussian soft label. (**f**) Machine learning flow chart. The upper part is the model training process. The lower part is the actual application process of the test.

Before establishing a neural network model, it is essential to collect a substantial amount of experimental data to create a reliable dataset. Figure 4a shows the raw voltage signals collected from the TENG at different rotation speeds. The dataset can be divided into training, validation, and test sets. The training set consists of data points, each with a length of 512 and an interval of 0.002 s, representing a total display time of 1 s. The vertical axis corresponds to the voltage values. In Figure 4b, we illustrate a Fourier transform on the data length of 128 data points at different rotation speeds to obtain the frequency domain information. The validation set data includes various rotation speeds of the TENG, which can be used as accurate target speeds for prediction, ultimately selecting the one with the highest accuracy. The algorithm model is then evaluated using the test set data to assess its prediction performance at different speeds. During the training process of machine learning algorithms, it may sometimes be necessary to normalize the data, as this can enhance training efficiency and improve the machine’s predictive performance on the target signal [50]. Figure 4c,d illustrates the specific details of the data processing with the voltage signals collected from the TENG. The entire dataset is divided into three parts, namely the training set, validation set, and test set. The length of the data collected in the experiment is 3200. The lengths of the training set, validation set, and test set are 725. An interval buffer of 512 data points is set between the three datasets. Whether it is the training set or the validation set, we use a sliding window strategy, taking 512 data points as the basic length unit and taking a sliding window every other point to divide the data into multiple samples that meet the model input requirements. Using sliding windows for data augmentation while enhancing the model’s generalization capability ensures that data starting from any time point is well-trained. Therefore, this division method not only ensures the integrity of the data but also ensures the reliability of the model training and evaluation process.

Since there may be outliers in the collected voltage signals, we tried different min-max normalization and z-score normalization methods and compared them. As shown in Figure S2, we found that z-score normalization can better monitor different speeds. Generally, when the neural network needs to input data 0–1, min-max normalization is usually used, while the z-score is more in line with the characteristics of physics itself. Therefore, we apply the z-score normalization method [50,51] to handle them.

(1) $$Z=\frac{X-\mu }{\sigma }\;\;\;\;\;\;\;\;\;\;\;\;\;\;\;\;\;\;\;\;$$

where Z is the normalized value, X are the original data, μ is the mean of the sample data, and σ is the standard deviation of the sample data.

In machine learning classification tasks, labels must be provided for the prediction process. One-hot encoding is an effective preprocessing method for data with distinct categorical values. It transforms each categorical value into a binary vector, ensuring that the model can accurately interpret non-numeric categorical features and avoid misinterpretations between values. As shown in Figure 4e, in the example of a one-hot label vector, the third value is one, indicating that the data belong to the third category with a 100% probability. Assuming there are n categories, one-hot encoding converts each category i into an n-dimensional vector $v_{i}$:

(2) $$v_{i}=\left[0,0,\ldots ,1,\ldots ,0\right]$$

where the value at position  i is one, indicating that the data belong to category i, while all other positions are zero.

However, in different studies, the specific nature of the data samples collected needs to be considered. For instance, in this study, predicting the rotational speed of an object is the machine’s prediction target. However, there is some correlation between different rotational speeds, so we softened the one-hot encoding by using a Gaussian function to distribute the target labels. This method generates a Gaussian soft label, which disperses the label probabilities of adjacent categories via the Gaussian function. Let the Gaussian softening function be defined as $G\left(i;\mu ,\sigma \right)$, The probability for label L can be expressed as

(3) $$L_{i}=G\left(i;\mu ,\sigma \right)=\frac{1}{\sqrt{2\pi }\sigma }e^{-\frac{{\left(i-\mu \right)}^{2}}{2\sigma^{2}}}$$

where μ is the center of the category and  σ is the standard deviation. The center of the category is the correct label corresponding to the rotation speed, and the standard deviation value is 10. This value provides a good balance where the Gaussian distribution is wide enough to ensure a smooth transition between adjacent classes without overlapping too much. Too small a value will result in too sharp boundaries, while too large a value will result in overly blurred class boundaries. For example, in the Gaussian soft label vector, the third value is 0.92, while the second and fourth values are 0.04, indicating a 92% probability that the data belong to the third category and a 4% probability for both the second and fourth categories. This softening approach better reflects the relationships between rotational speed categories and helps improve the model’s accuracy in prediction tasks. In the subsequent model training, we compared the results of the two encoding methods.

As shown in Figure 4f, the machine learning model mentioned above can be divided into two parts, namely the training process and application process. The main steps include data preprocessing, model training, verification, and final application testing. The details of each step are as follows:

(i) Data collection and processing: Two approaches were used to input the voltage signal data collected by the TENG. One approach was to completely input the original collected signal. The other approach was to input the signal after processing using the z-score normalization method. This can avoid the problem of the prediction results being affected by the presence of outliers in the collected voltage signal.
(ii) Offline training of the machine: First, the sample set is divided into three parts, namely a training set, validation set and test set. They are used to train the machine learning model and predict the TENG speed, respectively. Then, based on the sample set, the back propagation algorithm is used to optimize the training machine algorithm model.
(iii) Online prediction of the TENG: Based on the optimal machine learning algorithm model trained in step (ii), the processed real-time voltage signal can be used as input to perform online prediction of the real-time speed of the TENG. Finally, the machine can output the speed of the TENG at this moment to determine its status.

### 3.3. Machine Learning Monitoring Rotational Speed

In engineering practice, the demand for rotational speed measurement frequently arises across a wide range of scenarios and applications. Therefore, it is very necessary to use machine learning to monitor the speed of machinery. We mainly consider the three most classic machine learning algorithms, namely multilayer perceptron (MLP), long short-term memory networks (LSTM), and convolutional neural networks (CNNs) [52,53,54]. The specific details of the three machine learning algorithms used in this work are shown in Figure S1 (Supplementary Information). The results show that LSTM is the most efficient. The results of the confusion matrix for the classification predictions of three different machine learning algorithms, using different labels encoded in the voltage signal data, are shown in Figures S3 and S4 (Supplementary Information).

**Figure 5:**
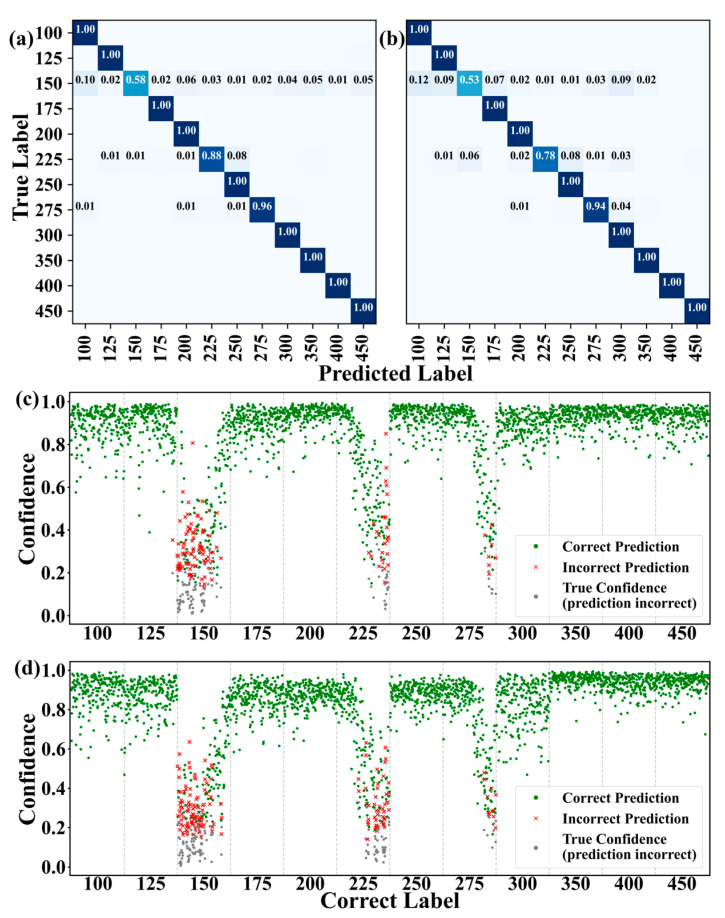
Machine learning monitoring the accuracy of rotational speed. (**a**,**b**) Classification results of LSTM model for signals at different rotational speeds using one-hot and Gaussian soft encoding for labels, respectively. The darker the color in the figure, the higher the accuracy. (**c**,**d**) Scatter plot of confidence of correct predictions when using different labeling methods for the LSTM model.

The classification and prediction results of LSTM for different rotational speeds with One-Hot and Gaussian soft encoding for labels are shown in Figure 5a,b. The LSTM results are slightly better than those of the MLP, although the classification at 150 rpm and 225 rpm remains worse than at other speeds. This indicates that the quality of raw data has a significant impact, and noise in the data collection process can influence the results. Therefore, the accuracy changes and stability improvements of the three algorithms after normalization were compared. In these tests, the input data length for all three algorithms was set to 512. To explain the results more clearly, we present a scatter plot to visualize the model predictions and actual rotation speeds (as shown in Figure 5c,d). The horizontal axis represents the index of the sample, with a total of 12 categories. The vertical axis represents the confidence of the prediction. The green points represent samples that are predicted correctly. The red crosses and gray points represent samples that are predicted incorrectly and the confidence of their true categories. The vertical dotted lines are used to separate different categories, and the labels at the bottom indicate the values corresponding to each category. As shown in Figure 5c, when the rotational speed is 150 rpm, there are more red points, and the prediction effect is poor. The corresponding accuracy is 0.58. A small number of red points also appear at 225 and 275 rpm, and the corresponding accuracy is 0.88 and 0.96, respectively. When the prediction is incorrect, the corresponding gray point distribution of the correct confidence is very close to the red points. Similarly, for Gaussian labels, it is also obvious that the speed is worse at 150 and 225 rpm. This also shows that there is a certain systematic error in the experimental process, which affects the results. Additionally, we evaluated the PR and F1 score curves of the LSTM model under different configurations, and the results are shown in Figure S5.

**Figure 6:**
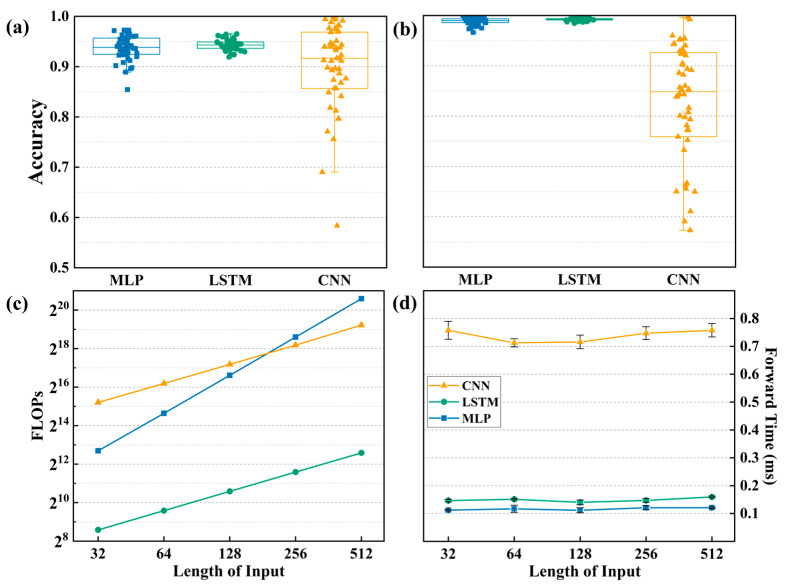
Machine learning monitoring the stability and computational cost statistics of rotational speed. (**a**,**b**) Scatter box plots for different algorithm models with and without normalization. (**c**,**d**) Computational cost statistics for different algorithms as the length of the training data varies.

Figure 6a shows a scatter of the correctness rate obtained for different models randomly implemented 50 times with different initial parameters when the data are not normalized. The accuracy of the MLP and LSTM mainly falls between 0.9 and 1.0, while the CNN’s data are more scattered, indicating poorer stability. As shown in Figure 6b, the scatter of the correctness rate was obtained for the same realization 50 times after the data had been normalized. The classification results of the LSTM and the MLP were close to one. Overall, the LSTM algorithm not only demonstrates better stability but also achieves higher accuracy in rotational speed classification predictions. In this work, the rotational speed is provided by the motor. The speed of the motor is controlled by adjusting the governor to provide a fixed speed for the TENG. The prediction accuracy of machine learning is actually calculated by comparing with the speed directly calibrated by the speed regulator. The prediction results of machine learning will depend on the collected signal. After the data are normalized, the prediction accuracy can reach more than 0.96. Generally, the accuracy (relative error) of conventional commercial speed sensors can reach 0.1–5%, and the absolute error is between 0.1 rmp and 100 rmp.

To intuitively compare the three algorithms, the computational cost variations with different training data lengths were statistically analyzed. Floating point operations (FLOPs) are mainly used to measure a model’s computational complexity, i.e., the amount of computation needed for inference or training. As shown in Figure 6c, the horizontal axis represents the data input length increasing from 32 to 512, with blue data points indicating the FLOPs for the MLP as the data length increases. We observe that the overall trend is linear growth. In comparison, the orange CNN data points show that initially, MLP’s computational cost is lower than the CNN’s, but as the input data length increases, MLP’s computational cost also increases, eventually surpassing the CNN’s slope. However, regardless of slope or values, the green LSTM data points show significantly lower computational costs than the other two algorithms. Figure 6d shows the comparison of computation times for the models under different data lengths. Although the computation time for all three algorithms remains relatively stable with changes in data input length, the computation time for the MLP and LSTM is significantly shorter than that of the CNN.

In summary, the LSTM algorithm demonstrates a clear advantage in both computational cost and time. LSTM is a recursive neural network that can effectively handle long sequence dependencies and gradient vanishing and exploding problems. It can remember previous information and predict current information. The advantage of LSTM is that it can process long sequence data and has good memory, but the training time is long. MLP is a feedforward neural network that can be used for tasks such as classification and regression. The advantage is that it has a fast training speed and can process a large number of features. However, for sequence data, the MLP does not perform as well as LSTM. However, the CNN is good at extracting high-level features from structured data. It is mainly used in image processing. In this work, the main processing is sequence data, so LSTM will perform better.

Furthermore, machine learning can detect any speed, which greatly improves the speed detection range. In many real scenarios, the data are affected by environmental noise. Even for the missing data and the influence of noise in the actual scene, corresponding processing can be performed to achieve good prediction results. Studies have shown that LSTM can introduce algorithms with denoising architectures. A deep neural architecture inspired by encoder–decoder language systems was implemented. A solution was found that can make high-precision predictions using models learned from training data of moving objects [55]. In addition, some studies have introduced self-powered, low-cost, and highly sensitive triboelectric sensors into the field of intention recognition and combined triboelectric sensors with deep learning algorithms, providing a promising solution for improving the safety of autonomous vehicles and the efficiency of intelligent transportation systems. In this complex and high-speed scenario, the recognition of emergency braking, sudden acceleration, normal braking, and normal acceleration is achieved [56]. Therefore, in future research, in addition to collecting as much and as diverse data as possible in experiments, we will also explore methods such as transfer learning or unsupervised learning to deal with the prediction challenges in the case of severe data loss or noise.

## 4. Conclusions

In order to better monitor the speed and health status of rotational machinery in practical applications, in this work, a prediction model for monitoring the speed of the TENG based on machine learning is proposed. The peak power of the rotational TENG proposed reaches up to 6.6 mW, which can instantly light up 65 LEDs connected in series. This study successfully achieved rotational speed classification prediction for a TENG by analyzing output signals. Based on the principle of establishing nonlinear relationships, other electrical signals that sufficiently represent the state features of the TENG can also provide data support for machine learning algorithms. Comparing the original training signal data with the normalized data reveals that normalization improves prediction accuracy. In addition, LSTM exhibits advantages in both computational cost and time. Hence, the results of training the output collected by the TENG using a machine learning algorithm show that LSTM can better predict and classify signals of different speeds. Furthermore, machine learning can detect any speed, which greatly improves the speed detection range. In future research, in addition to collecting as much and as diverse data as possible during experiments, methods such as transfer learning or unsupervised learning could be explored to address prediction challenges in cases of severe data loss. Furthermore, machine learning can detect any speed, which greatly improves the speed detection range.

## 5. Details of the Experiment

### 5.1. Rotating TENG

To stimulate the TENG to generate voltage signals, a data collection platform was constructed. As shown in Figure 2, the data acquisition platform mainly consists of the TENG, a PC, an oscilloscope (Hantek DSO5102P, Hantek Electronics, Qingdao, China), and an electrometer (Keithley 6514, Keithley Instruments, Cleveland, OH, USA). First, two acrylic plates measuring 30 cm × 30 cm × 0.5 cm were cut. One acrylic plate was placed above the motor and fixed to the bottom porous plate with bolts and nuts. The other plate was also fixed to the lower acrylic plate with bolts and nuts, serving to secure the stator of the disk-shaped TENG. The motor was positioned between the porous plate and the acrylic plate to provide excitation for the disk-shaped TENG. Two acrylic plates, each with a diameter of 18 cm and a thickness of 0.5 cm, were cut to serve as the base of the disk-shaped TENG, with a small hole of 14 mm diameter left at the center of the base. The friction materials of the TENG were copper and PTFE films. A circular copper film with a diameter of 16.6 cm and a thickness of 50 µm was divided into eight equal parts and evenly adhered to the base, serving as the stator of the disk-shaped TENG. Two wires connected four sections of the copper film to create the two output electrodes. Four PTFE films of the same thickness and size were adhered to equally sized foam boards, which were then evenly spaced and attached to another acrylic base, serving as the rotor of the disk-shaped TENG. Two flange seats connected the linear optical shaft to the motor shaft, allowing the linear optical shaft to pass through the small hole of the disk-shaped TENG, with the flange seat securing the rotor and linear optical shaft. By adjusting the motor to generate different rotational speeds, varying outputs from the disk-shaped TENG were produced. A digital oscilloscope was used to collect the output voltage signals. The electrometer was controlled via a PC to gather short-circuit current and transferred charge data. At maximum speed, different resistors were connected to measure peak current, allowing for the calculation of peak power.

### 5.2. Training of Machine Learning Models

In this work, the experimental hardware platform comprised an Intel(R) Core(TM) i7-11800H CPU (Intel Corporation, Santa Clara, CA, USA) and an NVIDIA GeForce RTX 3070 Laptop GPU(NVIDIA Corporation, Santa Clara, CA, USA), while the software platform was based on Windows 10 (Microsoft Corporation, Redmond, WA, USA). The main development environment used Python 3.10 (Python Software Foundation, Wilmington, DE, USA) and PyTorch 2.1.0 (Meta Platforms, Inc., Menlo Park, CA, USA). During model training, the number of epochs was set to 1000 for the MLP and CNN models and 200 for the LSTM model (we select the model with the best performance on the validation set in one training session for testing to ensure a fine evaluation of the model performance). A batch size of 512 was selected so that 512 samples were used for each gradient update. The initial learning rate was set to $1\times 10^{-3}$, with the CosineAnnealingLR scheduler applied to progressively decrease the learning rate, with a minimum learning rate of $\eta_{\min}=1\times 10^{-5}$ and the $T_{\max}$ set to correspond to the total number of model updates to stabilize convergence. The AdamW (from PyTorch 2.1.0) optimizer was chosen with a weight decay of $1\times 10^{-3}$. For classification tasks, CrossEntropyLoss was employed as the loss function. Any other parameters not specifically mentioned were left at their default values.

## Data Availability

The data that support the findings of this study are available from the corresponding author upon reasonable request.

## Supplementary Materials

The following supporting information can be downloaded at: https://www.mdpi.com/article/10.3390/s25082533/s1, Figure S1: Diagram of machine learning algorithms. Figure S2: Comparison of results of different normalization in LSTM algorithm. Figure S3: Confusion matrices of classification prediction results for three different machine learning algorithms applied to voltage signal data using different label encoding methods, without normalization. Figure S4: Confusion matrices of classification prediction results for three different machine learning algorithms applied to normalized voltage signal data using different label encoding methods. Figure S5: The PR and F1-score curves under different configurations of the LSTM model.
