# Supplementary material for: Rotational Triboelectric Nanogenerator with Machine Learning for Monitoring Speed
Source: Sensors (Basel). 2025 Apr 17;25(8):2533. doi: 10.3390/s25082533 (PMC12031442; doi:10.3390/s25082533)
Supplement: Supplementary file 1 [file sensors-25-02533-s001.zip › sensors-3555785-supplementary.pdf]

Supplementary Information

Rotational Triboelectric Nanogenerator with Machine Learning for Monitoring Speed

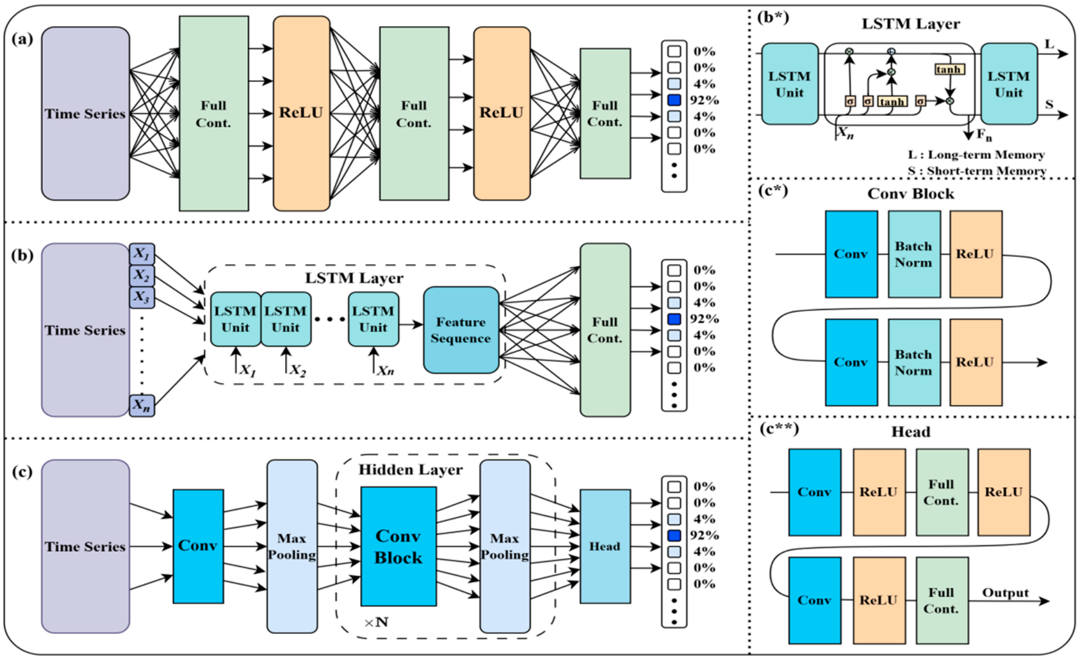

Figure S1. **Diagram of machine learning algorithms. (a) The overall procedural architecture of the MLP model, which incorporates two hidden layers and one output layer; the hidden layers include a linear function and an activation function. (b) The overall procedural architecture of the LSTM model. (b*) The operational flow of each cell unit within the LSTM layer, where data is sequentially input into the model, cyclically passing through the forget gate, memory gate, and output gate to yield features. (c) The overall procedural architecture of the CNN model. (c*) The convolution block within the hidden layers, where the input data undergoes two iterations of convolution, batch normalization, and activation processes. (c**) The head output layer, where the input features pass through convolution, activation, fully connected layers, and further activation for mixed-dimensional detection, ultimately resulting in convolution, activation, and fully connected output.**

The specific details of the three machine learning algorithms used in this work are shown in Figure S1. Figure S1(a) is the overall process architecture of the MLP model, which includes two hidden layers and one output layer, where the hidden layer includes a linear function and an activation function. The linear transformation in the hidden layers:

$$Y_i = W_i a_{i-1} + b_i$$

and the activation function, typically a ReLU:

$$a_i = \max(0, Y_i)$$

.

The hidden layer node for this MLP is $(2n, n)$, with the output layer node count set to$(n/2)$. The n denotes the number of input data nodes and Y_i represents the linear output of the i-th node in the hidden layer. The matrix W_i denotes the weights associated with the i-th node, which determine the influence of the inputs. The term a_{i-1} signifies the output from the previous layer, whether it be the input layer or another hidden layer. Lastly, b_i is the bias term for the i-th node, providing additional flexibility for the model to fit the data effectively.

Figure S1(b) shows the overall procedural architecture of the LSTM model, which includes a unidirectional LSTM layer designed to extract features that are subsequently fed into an output layer. The operational flow within each cell unit of the LSTM layer is depicted in Figure S1(b*), where sequential data is input into the model, traversing cyclically through the forget gate, memory gate, and output gate to yield features. The hidden node count for this LSTM is designated as$(n/2)$.

Figure S1(c) shows the overall process architecture of the CNN model, which features a unique mixed-dimensional detection design. After the data passes through the initial layer of convolution, it is processed through multiple hidden layers for feature extraction. Finally, the data reaches the head output layer, where these extracted features are synthesized for the final output. The input channel number is 1, the initial convolution kernel size is 3×1 with a stride of 1 and symmetric padding (padding=1); the feature dimension is gradually expanded through three stages (4 to 8 to 16 channels), with each stage containing one residual block. The fully connected layers adopt a phased dimensionality reduction strategy (16 to 8 to 1), and the final output dimension

aligns with the classification target. Figure S1(c*) illustrates the convolution block within the hidden layer, where the input data undergoes two convolutions, followed by batch normalization and activation. Figure S1(c**) depicts the head output layer, where the input features are further processed through convolution, activation, fully connected layers, and additional activation, ultimately resulting in a fully connected output. The batch normalization equation of the CNN can be written as follows,

$$\hat{X} = \frac{X - \mu}{\sqrt{\sigma^2 + \epsilon}}$$

$$Y = \gamma \hat{X} + \beta$$

where $\hat{X}$ is the normalized output from batch normalization. The variable X denotes the input feature map, μ is the mean, σ^2 is the variance, ϵ is a small constant, and γ and β are learnable parameters for scaling and shifting. These components work together to enable the CNN to effectively learn and capture hierarchical features from the input data.

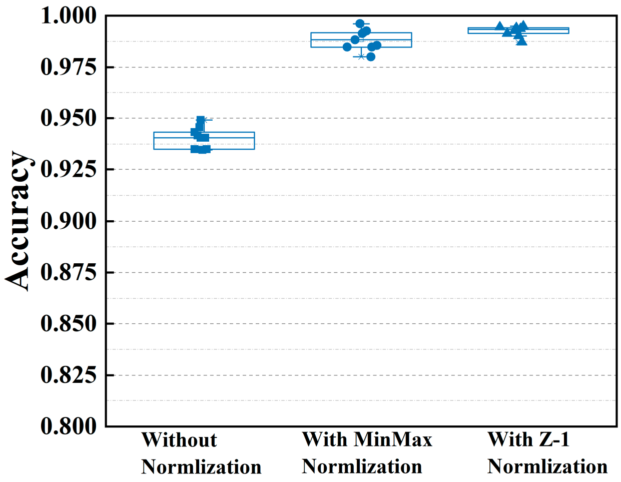

Figure S2. Comparison of results of different normalization in LSTM algorithm.

In this work, we tried different Min-Max normalization and Z-Score normalization methods and compared them. As shown in Figure S2, we found that Z-Score normalization can better monitor different speeds. Generally, when the neural network needs to input data 0-1, Min-Max normalization is usually used, while Z-score is more in line with the characteristics of physics itself. Therefore, we chose to use z-score normalization in this work.

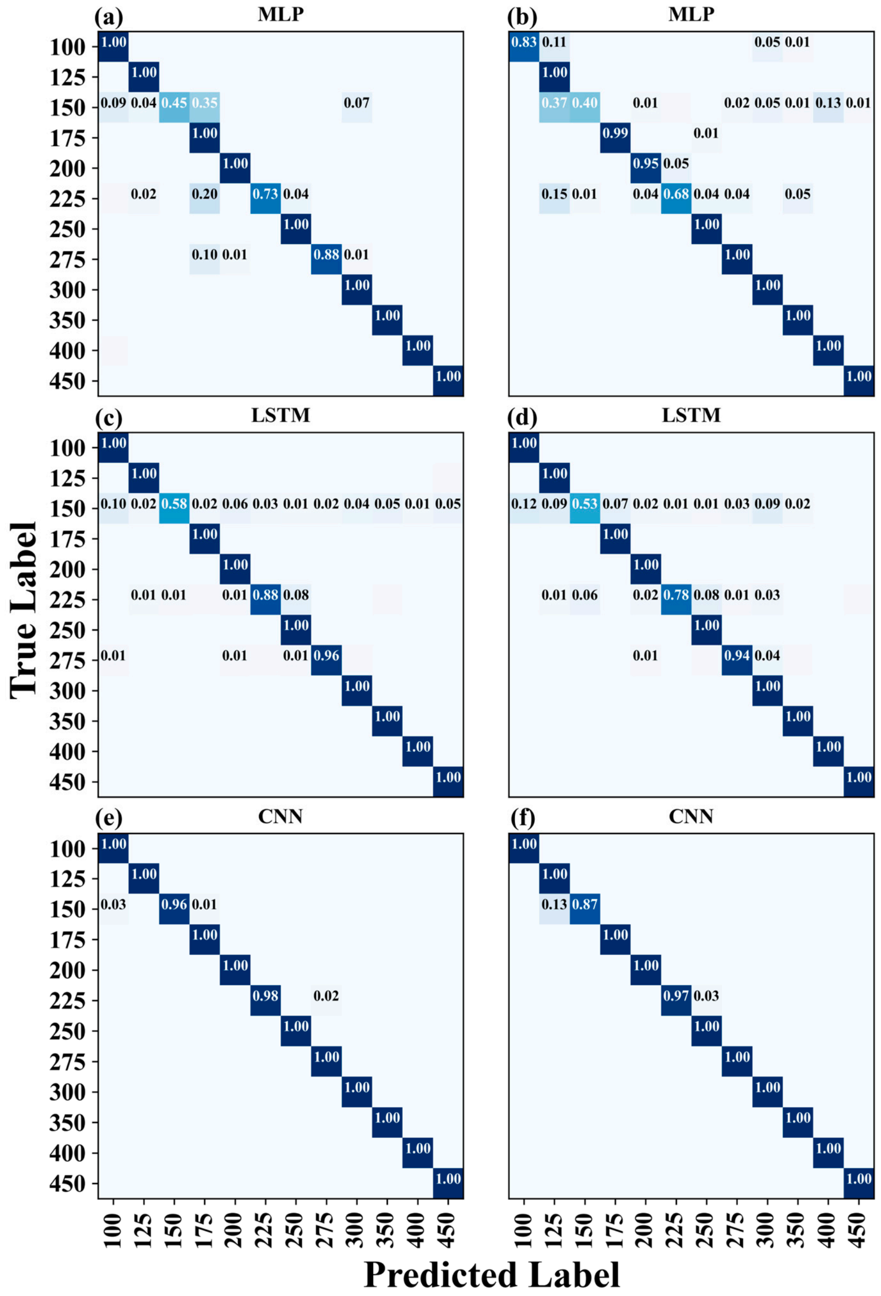

Figure S3. Confusion matrices of classification prediction results for three different machine learning algorithms applied to voltage signal data using different label encoding methods, without normalization. (a-b) Classification results of MLP model for signals at different rotational speeds using One-Hot and Gaussian soft encoding for labels, respectively. (c-d) Classification results of LSTM model for signals at different rotational speeds using One-Hot and Gaussian soft encoding for labels, respectively. (e-f) Classification results of CNN model for signals at different rotational speeds using One-Hot and Gaussian soft encoding for labels, respectively.

Figure S3 shows the classification and prediction results of MLP, LSTM, and CNN models using One-Hot and Gaussian soft encoding for labels at different rotational speeds. The input data for this Fig. consists of raw data that has not been normalized. As seen in Figure S3(a-b), with two different label settings, the MLP algorithm can accurately classify the signals, except for lower accuracy at 150 rpm and 225 rpm. Figure S3(c-d) show the results of the LSTM algorithm under the same conditions. The LSTM results are slightly better than those of MLP, although the classification at 150 rpm and 225 rpm remains worse than at other speeds. This indicates that the quality of raw data has a significant impact, and noise in the data collection process can influence the results. Figure S3(e-f) display the classification results of the CNN algorithm under the same conditions, and it's evident that CNN performs much better than MLP and LSTM, with accuracy over 0.95, except for 150 rpm in Figure S3(f), which has an accuracy of 0.87. Our analysis suggests that CNN inherently includes a normalization process, which improves overall classification by preprocessing the raw data and reducing noise's effect. Given that normalization preprocessing may significantly affect the results, we next apply normalization to the raw data and then use the three models for training and prediction.

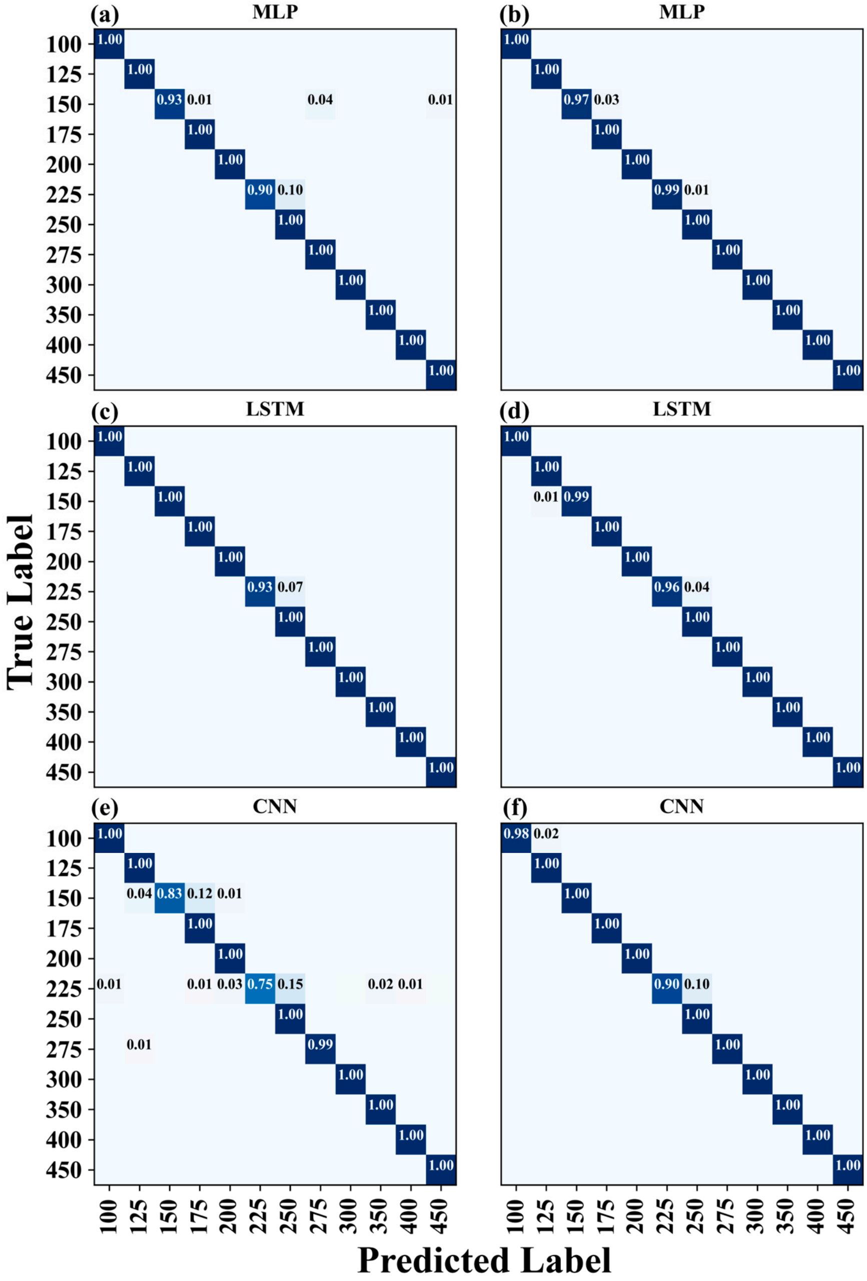

Figure S4. **Confusion matrices of classification prediction results for three different machine learning algorithms applied to normalized voltage signal data using different label encoding methods. (a-b) Classification results of MLP model for signals at different rotational speeds using One-Hot and Gaussian soft encoding for labels, respectively. (c-d) Classification results of LSTM model for signals at different rotational speeds using One-Hot and Gaussian soft encoding for labels, respectively. (e-f) Classification results of CNN model for signals at different rotational speeds using One-Hot and Gaussian soft encoding for labels, respectively.**

As shown in Figure S4, we performed training tests with the normalized data for all three algorithms. Comparing with Figure S4 (a-d), we find that normalization greatly improves the prediction results for MLP and LSTM. In contrast, the results for CNN are not as good, as seen in Figure S4 (e-f). Because CNN already incorporates normalization. And applying it again weakens some of the data’s inherent characteristics, leading to less noticeable improvements in training results and a marked decline in CNN's predictive performance.

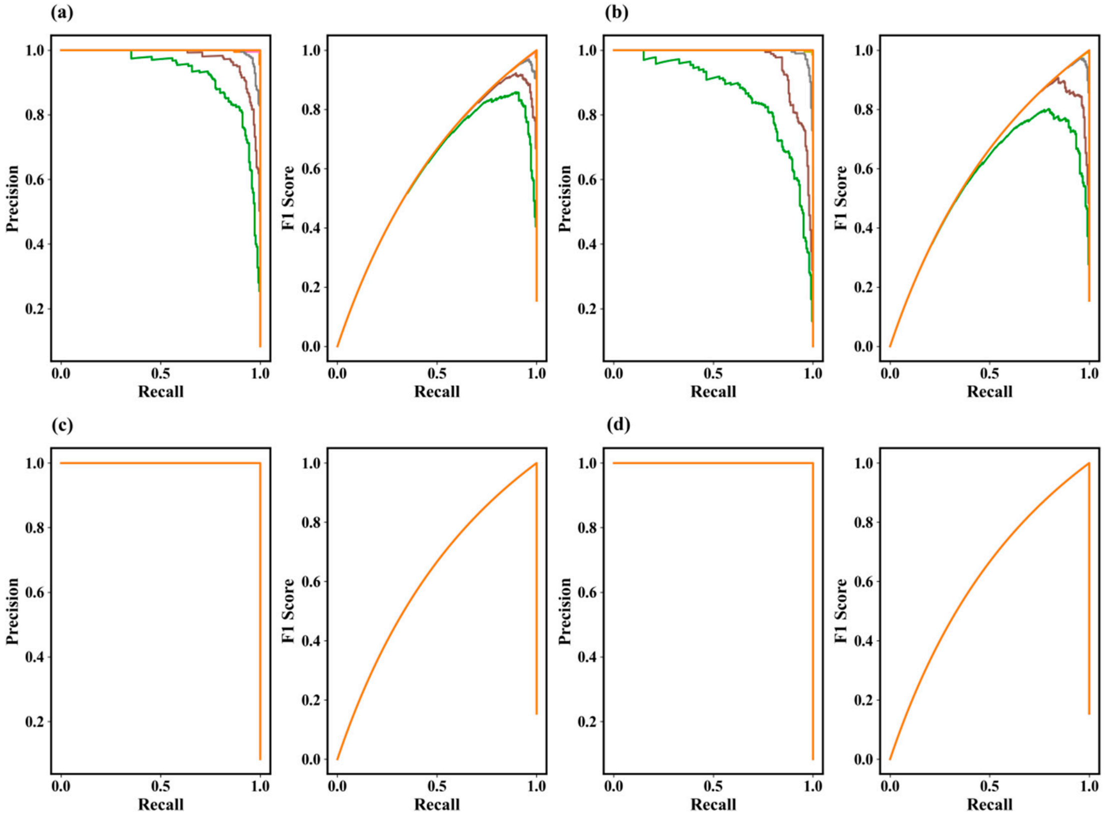

Figure S5. The PR and F1-score curves under different configurations of the LSTM model: (a) shows the baseline performance of the LSTM model without any normalization or application of Gaussian soft labels. The PR curve (left) and F1-score curve (right) reflect the model's performance under these basic conditions. The different colored lines in the PR curve represent different classes being predicted. (b) illustrates the impact of incorporating Gaussian soft labels on the model's performance while keeping the input features unnormalized and used Gaussian Soft Label. (c) illustrates the input features are normalized, but Gaussian soft labels are not used. (d) presents the performance of the LSTM model with both normalization and Gaussian soft labels applied.

Figure S5 presents a detailed evaluation of the LSTM model's performance under various configurations using Precision-Recall (PR) and F1-score curves. These metrics are crucial for understanding the model's effectiveness in classification tasks. The PR curve illustrates the relationship between precision (the proportion of correctly predicted positive instances among all predicted positives) and recall (the proportion of correctly predicted positive instances among all actual positives) at different thresholds. The F1-score curve displays the F1-score, which is the harmonic mean of precision and recall, providing a single, balanced measure of performance against the recall threshold. By examining these four sets of curves within Figure S5, we can systematically analyze the specific contributions and interactions of data normalization and Gaussian soft labeling techniques on the predictive capabilities of the LSTM model for this task.
